# Analysis of the Virulence of an Atypical Enteropathogenic *Escherichia coli* Strain *In Vitro* and *In Vivo* and the Influence of Type Three Secretion System

**DOI:** 10.1155/2014/797508

**Published:** 2014-04-28

**Authors:** Suely C. F. Sampaio, Fabiana C. Moreira, Ana M. A. Liberatore, Mônica A. M. Vieira, Terezinha Knobl, Fabiano T. Romão, Rodrigo T. Hernandes, Claudete S. A. Ferreira, Antônio P. Ferreira, Aloísio Felipe-Silva, Rita Sinigaglia-Coimbra, Ivan H. J. Koh, Tania A. T. Gomes

**Affiliations:** ^1^Departamento de Microbiologia, Imunologia e Parasitologia, Universidade Federal de São Paulo, Escola Paulista de Medicina, Rua Botucatu 862, 3rd floor, Vila Clementino, 04023-062 São Paulo, SP, Brazil; ^2^Departamento de Cirurgia, Universidade Federal de São Paulo, Escola Paulista de Medicina, Rua Pedro de Toledo 781, 9th floor, Vila Clementino, 04039-032 São Paulo, SP, Brazil; ^3^Departamento de Ornitopatologia, Faculdade de Medicina Veterinária, Universidade de São Paulo, Avenida Prof. Dr. Orlando Marques de Paiva 87, Cidade Universitária, 05508-900 São Paulo, SP, Brazil; ^4^Departamento de Microbiologia e Imunologia, Instituto de Biociências, UNESP, Distrito de Rubião Junior s/n, Botucatu, 18618-970 São Paulo, SP, Brazil; ^5^Fleury Medicina e Saúde, Avenida General Valdomiro de Lima 508, 04344-903 São Paulo, SP, Brazil; ^6^Centro de Microscopia Eletrônica, Universidade Federal de São Paulo, Escola Paulista de Medicina, Rua Botucatu 862, 1 st floor, 04023-062 São Paulo, SP, Brazil

## Abstract

Atypical enteropathogenic *Escherichia coli* (aEPEC) inject various effectors into intestinal cells through a type three secretion system (T3SS), causing attaching and effacing (A/E) lesions. We investigated the role of T3SS in the ability of the aEPEC 1711-4 strain to interact with enterocytes *in vitro* (Caco-2 cells) and *in vivo* (rabbit ileal loops) and to translocate the rat intestinal mucosa *in vivo*. A T3SS isogenic mutant strain was constructed, which showed marked reduction in the ability to associate and invade but not to persist inside Caco-2 cells. After rabbit infection, only aEPEC 1711-4 was detected inside enterocytes at 8 and 24 hours pointing to a T3SS-dependent invasive potential *in vivo*. In contrast to aEPEC 1711-4, the T3SS-deficient strain no longer produced A/E lesions or induced macrophage infiltration. We also demonstrated that the ability of aEPEC 1711-4 to translocate through mesenteric lymph nodes to spleen and liver in a rat model depends on a functional T3SS, since a decreased number of T3SS mutant bacteria were recovered from extraintestinal sites. These findings indicate that the full virulence potential of aEPEC 1711-4 depends on a functional T3SS, which contributes to efficient adhesion/invasion *in vitro* and *in vivo* and to bacterial translocation to extraintestinal sites.

## 1. Introduction

Atypical enteropathogenic* Escherichia coli* (aEPEC) are emerging agents of diarrhea. They differ from typical EPEC (tEPEC) strains mainly by the absence of the EAF (EPEC adherence factor) plasmid [1, 2]. Like tEPEC, aEPEC strains inject various effector proteins into enterocytes through a type three secretion system (T3SS) leading to the formation of attaching-effacing (A/E) lesions [3–5]. The assembly of T3SS is dependent on an ATPase encoded by* escN*, and consequently,* escN* mutants are incapable of assembling or injecting effector proteins via T3SS into the host cell cytoplasm [6]. Tir (translocated intimin receptor) is a T3SS-dependent effector protein, which is inserted in the eukaryotic cell membrane and interacts with an EPEC outer membrane adhesive protein (intimin) [7]. Tir-intimin interaction leads to the establishment of A/E lesions [8]. Many other T3SS-dependent effector proteins, such as Map (mitochondrial-associated protein) and EspF, have important roles in aEPEC pathogenesis. These proteins have redundant functions and can cause epithelial barrier disruption by interacting with tight junctions, leading to cell death by apoptosis [9, 10].

Bacterial translocation (BT) is defined as the phenomenon by which live bacteria and/or their products cross the intestinal barrier reaching normally sterile extraintestinal sites, such as the liver, spleen, and mesenteric lymph nodes (MLN). The translocation of certain indigenous bacteria from the gastrointestinal tract to the MLN and various organs had been previously demonstrated in a gnotobiotic mouse model [11]. There is much circumstantial proof that translocation is associated with an increased occurrence of postoperative septic complications, and* E. coli* has been reported to be one of the most common BT-associated organisms isolated from surgical patients with postoperative sepsis [12, 13]. In humans, one of the most well-studied translocation events is that observed in cirrhotic patients with spontaneous bacterial peritonitis (SBP) [14].

We recently demonstrated that an aEPEC strain (1711-4) is able to invade and induce inflammatory responses in intestinal Caco-2 cell lines [15]. This strain is also able to invade these cells* in vitro* and to escape from the intracellular compartment on the basolateral side [16]. In addition, we have demonstrated that in an experimental BT-rat model, aEPEC 1711-4 can reach the MLN, liver, and kidneys [17]. We also showed that aEPEC 1711-4 infected-animals had intestinal mesenteric microcirculation injury and systemic hypoperfusion similar to those observed with the virulent murine* E. coli *strain R6 [17, 18]. In the BT-rat model, the latter strain was recovered from the MLN, liver, and spleen and impaired mesenteric microcirculation [19].

The role of T3SS-dependent effector proteins in the ability of aEPEC to invade and persist in the intracellular compartment* in vitro* and to cross the intestinal barrier* in vitro* and* in vivo* is not yet established. The objective of this study was to determine the role of T3SS in the ability of aEPEC 1711-4 to invade and persist inside polarized intestinal cells* in vitro* (Caco-2 cells), to promote A/E lesions and invade* in vivo* (rabbit ligated ileal loop model), and to pass through the intestinal barrier in an* in vivo* experimental model (bacterial translocation model).

## 2. Materials and Methods

### 2.1. Ethics Statement

This study was carried out in strict accordance with the recommendations of the Ethical principles of the Sociedade Brasileira de Ciência em Animais de Laboratório (COBEA). The protocol was approved by the Committee on Research Ethics of the Universidade Federal de São Paulo (Permit number: 0235/12). All surgery was performed under Telazol anesthesia (rabbits) or xylazine hydrochloride plus ketamine hydrochloride (rats), and all efforts were made to minimize suffering.

### 2.2. Bacterial Strains and Growth Conditions

aEPEC 1711-4 (serotype O51:H40), which was isolated from a child with diarrhea in the city of São Paulo [20], an isogenic mutant deleted in the* escN* gene (1711-4 Δ*escN*), and a complemented mutant 1711-4 Δ*escN* (pEscN) were used. The nonpathogenic* E. coli* strain HS was used as a negative control (Table 1). The strains were cultivated overnight at 37°C in 5 mL of Luria-Bertani (LB) broth. The 1711-4 Δ*escN* and the 1711-4 Δ*escN* (pEscN) strains were cultivated in LB broth containing zeocin (60 *μ*g mL^−1^) and zeocin-chloramphenicol (30 *μ*g mL^−1^), respectively.

### 2.3. Construction of an Isogenic escN Deficient Mutant of aEPEC 1711-4 and Mutant Complementation

The* escN*-deficient mutant was constructed by homologous recombination using the Lambda Red system as previously described [15, 22]. Primers ESCN.zeo5 and ESCN.zeo3 were used to amplify the zeocin resistance gene (Table 2). The amplified product was electroporated into the 1711-4 strain containing the pKOBEG-Apra plasmid. Transformants were selected on LB agar containing zeocin (60 *μ*g mL^−1^). Deletion of the* escN* gene was confirmed using primers ESCN.verf5 and ESCN.verf3, targeting regions flanking this gene (Table 2). For complementation, the plasmid pEscN (pACYC184 vector carrying the* escN* gene) was electroporated into 1711-4 Δ*escN *and transformants were selected on LB agar containing chloramphenicol (30 *μ*g mL^−1^) [6].

### 2.4. Fluorescent-Actin Staining (FAS) Test in HeLa Cells

This test allows an indirect evaluation of the pathogen's ability to induce A/E lesions evidenced by actin nucleation underneath the site of intimate bacterium-enterocyte interaction [23]. Bacteria were grown in 5 mL of LB broth for approximately 18 h, in ambient air, at 37°C. Caco-2 cells were grown in 24-well plates (Corning) containing glass coverslips. They were cultivated in Dulbecco's modified Eagle's minimal essential medium (DMEM) supplemented with 10% fetal bovine serum (FBS) in a 5% CO_2_ atmosphere at 36 ± 1°C. Cells were grown up to 80% confluence. Cells were then washed three times with phosphate-buffered saline (PBS) before DMEM supplemented with 10% FBS containing 40 *μ*L of bacterial suspension (~10^8^ CFU mL^−1^) was added. Three hours after infection, cells were washed with PBS before they were fixed with 3% formaldehyde and permeabilized with 1% Triton X-100 for 4 min. Cells were washed with PBS and then incubated with PBS containing 5 *μ*g/mL fluorescein isothiocyanate (FITC)-conjugated phalloidin (Sigma-Aldrich) for 20 min in a dark chamber. Cells were then washed three times with PBS every 10 min. Coverslips were removed, dried, and placed inverted onto glass slides containing 10 *μ*L of 80% glycerol in PBS. Preparations were examined under fluorescence microscopy.

### 2.5. Infection of Caco-2 Cell Monolayers

Monolayers of postconfluent and differentiated Caco-2 cells were infected with ~1 ×  10^7^ colony forming units (CFU) mL^−1^ in each well of a 6-well cell culture plate. The number of cell-associated bacteria was determined three hours after infection. Cells were washed with phosphate-buffered saline pH 7.2 (PBS) before they were lysed with 1% (v/v) Triton X-100. Bacterial suspensions were plated on LB agar to determine the number of CFU. Bacterial invasion and persistence were assessed using gentamicin (100 and 10 *μ*g mL^−1^, resp.) to kill extracellular bacteria before eukaryotic cell lysis for determination of the number of viable bacteria. All tests were performed twice in triplicate. The percentage of bacteria recovered after 48 h (persistence index) was calculated taking the number of CFU at three hours as 100% [15].

### 2.6. Rabbit Ligated Ileal Loop Model

Prior to the assays, New Zealand White rabbits (weighing 1.8 to 2.5 kg and 4 to 8 weeks of age) were examined for the presence of A/E lesion-producing* E. coli *by PCR using primers that identify the* eae* gene [24]. All bacterial strains were tested in three animals. Rabbits were fed only 10% (w/v) glucose solution for 48 h prior to the test. Rabbits were anesthetized with an intramuscular injection of Telazol (Fort Dodge Animal Health, Iowa, USA) (0.2 mL kg^−1^) and sedated with Nilperidol (0.3 mL kg^−1^, Cristália, São Paulo, Brazil). Antisepsis with 70% (v/v) ethanol was performed after shaving the abdomen. The mid-ileum was exposed by a midline laparotomy, and through a small incision made on the ileum wall, the distal portion was gently washed using a syringe with sterile saline to minimize the presence of luminal feces and resident microbiota. Immediately afterwards, five separated ileum segments, measuring 5 cm long and 3 cm apart, were constructed by ligatures, and 0.3 mL of a bacterial suspension (1 × 10^8^ CFU mL^−1^) in sterile LB broth was injected into each ligated loop using a 25-gauge needle. The ileum was then returned to the abdominal cavity, and the peritoneal membrane and the abdominal wall were sutured. Animals were kept fasting for eight or 24 h and then were sacrificed with 3% (w/v) pentobarbital and zolazepam hydrochloride (0.4 mL kg^−1^). Ileal fragments including the whole intestinal wall were excised and fixed in 3% (w/v) glutaraldehyde in 0.1 M sodium cacodylate buffer, pH 7.2, for electron microscopy procedures.

### 2.7. Transmission Electron Microscopy (TEM)

After fixing with 2.5% (v/v) glutaraldehyde for 24 h at 4°C, the ileal fragments were rinsed with 0.1 M cacodylate buffer, pH 7.4, and postfixed in 1% (w/v) osmium tetroxide. Specimens were then exposed to a graded ethanol series and to propylene oxide. After embedding in Araldite resin and polymerization at 60°C for 48 h, ultrathin sections were stained with 2.0% (w/v) aqueous uranyl acetate and 2.5% (w/v) lead citrate. The specimens were then examined under a transmission electron microscope (LEO 906E; Zeiss) at 80 kV.

### 2.8. Histopathological Analyses

Transverse segments of rabbit ileum were fixed in buffered formalin before they were processed and embedded in paraffin. Sections were stained with hematoxylin-eosin before they were examined by a pathologist without previous knowledge of the details of the rabbit ileal loop experiments. Microscopy was carried out with a Zeiss microscope model Axio Lab.A1.

### 2.9. Bacterial Translocation Assays

Prior to the assays, adult female Wistar-EPM rats weighing 200–250 g (*n* = 11/bacterial strain) were examined for the presence of A/E lesion-producing* E. coli* as described above. Animals received rat chow and water* ad libitum*, and 24 h before the experiments, animals were fasting but had free access to water. During the experiments, animals were kept under anesthesia (xylazine hydrochloride plus ketamine hydrochloride (1 : 4), 0.1 mL per 100 g body weight, intramuscular). After antisepsis with 70% (v/v) ethanol and midline laparotomy, the terminal ileum was ligated, the second portion of the duodenum was repaired, and an oroduodenal catheter was inserted. Subsequently, an inoculum of 10^10^ CFU mL^−1^ (5 mL per 100 g body weight) was injected through the catheter and confined to the entire small bowel segment by the duodenum ligature. In six animals, saline was used instead of bacterial suspension (sham). The abdominal wall was closed with stitches after catheter removal. Sodium dipyrone (25 mg per kg body weight) was used for analgesia. After a period of two hours, animals were again subjected to laparotomy under anesthesia and samples were collected for analysis: one milliliter of blood from the inferior cava vein, MLN, spleen, and liver. Upon completion of the procedures, the animals were sacrificed by sectioning the aorta, still under anesthesia. Organs were weighed separately, crushed, macerated, and suspended in sterile saline, and the filtrate was plated on MacConkey agar to determine the number of translocated bacteria. Twenty-four hours after incubation in ambient air at 37°C, the translocated bacteria in the plate were counted and CFU/g/compartment were determined [25].

### 2.10. Statistical Analyses

Data were analyzed using Prism program version 5.03 from GraphPad Software. Analysis of variance (ANOVA) with Bonferroni post hoc test was applied to evaluate all results. ANOVA and Fisher's exact test were used for analysis of the BT results.

## 3. Results

### 3.1. T3SS Mutant of aEPEC 1711-4 (1711-4 ΔescN) Is Unable to Cause A/E Lesion and Is Required for Efficient Association of aEPEC 1711-4 with Differentiated Caco-2 Cells* In Vitro* While escN Complementation Restores These Features

**Figure 1:**
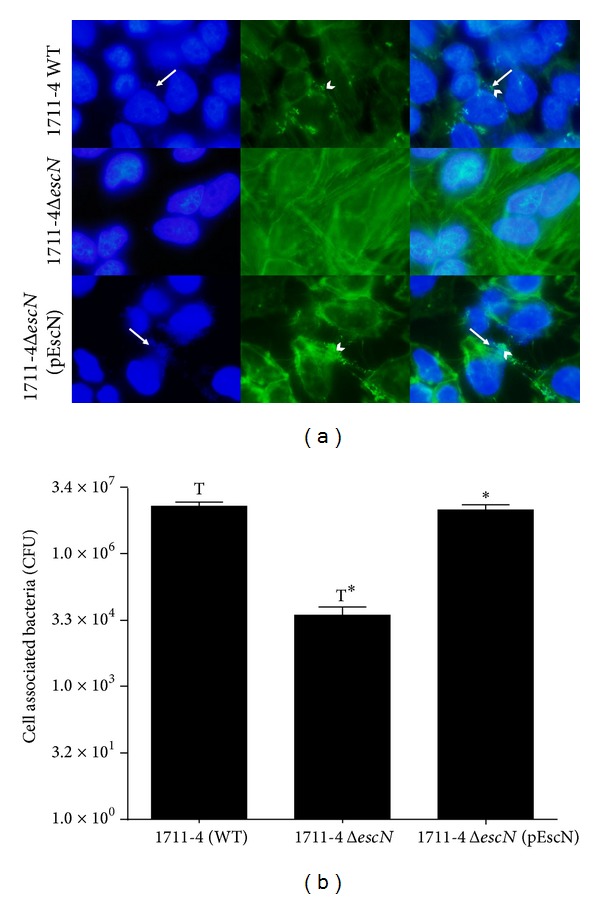
Lack of T3SS renders aEPEC 1711-4 unable to aggregate actin in HeLa cells and association with Caco-2 cells is decreased in the absence of T3SS. The ability of the wild-type strain 1711-4, its isogenic mutant deficient in* escN*, and the complemented mutant 1711-4 Δ*escN* (pEscN) as well to promote actin aggregation* in vitro* (evidence of A/E lesion formation) was examined by FAS. For actin accumulation, cells were stained with fluorescein isothiocyanate (FITC)-conjugated phalloidin (green), and bacterial (white arrow) and HeLa cell DNA was stained with DAPI (blue). No actin nucleation was observed with 1711-4 Δ*escN* mutant, whereas the ability of the complemented mutant to induce actin nucleation was restored (white arrowhead) (Figure 1(a)). Bacterial association was evaluated six hours after infection of differentiated Caco-2 cells. The number of viable bacteria recovered from cells infected with 1711-4 Δ*escN *mutant (~4.0×10^4^ CFU/well) was significantly lower compared with the wild-type strain (1.2 × 10^7^ CFU/well) (*P* < 0.05). *The association capacity of the* escN* mutant was restored in the 1711-4 Δ*escN* (pEscN) complemented strain and no statistically significant difference was observed when compared to the wild-type strain aEPEC 1711-4 (*P* > 0.05) (Figure 1(b)).

The ability of the wild-type strain 1711-4, its T3SS isogenic mutant (deficient in* escN*), and complemented mutant 1711-4 Δ*escN* (pEscN) as well to cause A/E lesions* in vitro* was evaluated using the FAS test. As expected and in contrast to the wild-type strain, no actin nucleation was observed with the 1711-4 Δ*escN* mutant, whereas the ability of the complemented mutant to induce actin nucleation was restored as in Figure 1(a). Although we have obtained the same results in Caco-2 cells, the presence of microvilli in polarized cells hampered the generation of a sharp image.

Bacterial association was evaluated three hours after infection of Caco-2 cells. The number of viable bacteria recovered from cells infected with the 1711-4 Δ*escN *mutant was on average 4 × 10^4^  CFU/well. This number was significantly lower (*P* < 0.05) than that obtained with the wild-type strain (1.2 × 10^7^ CFU/well). The association capacity of the* escN* mutant was restored in the 1711-4 Δ*escN* (pEscN) complemented strain, with no statistically significant difference (*P* > 0.05) when compared to the wild-type strain Figure 1(b).

### 3.2. T3SS Mutant (1711-4 ΔescN) Has a Decreased Ability to Invade Differentiated Caco-2 Cells but Persist Intracellularly

Gentamicin protection assays were used to evaluate bacterial invasion, while persistence was evaluated 48 h after infection. The mean number of CFU/well recovered from Caco-2 cells infected with the wild-type strain was approximately 30-fold higher than that observed with the 1711-4 Δ*escN* mutant. In addition, the mean CFU number obtained with the complemented mutant 1711-4 Δ*escN* (pEscN) was approximately 27-fold higher than that observed with the 1711-4 Δ*escN* mutant (Table 3). These differences were statistically significant (*P* < 0.05).

For bacterial persistence evaluation, monolayers were washed with PBS three hours after infection and incubated with DMEM containing gentamicin (10 *μ*g mL^−1^) to eliminate extracellular bacteria. The number of CFU recovered 48 h after infection with the 1711-*4 ΔescN* mutant was approximately 7-fold lower (*P* < 0.05) than that observed with the wild-type strain, while the complemented mutant 1711 Δ*escN* (pEscN) showed restored ability to persist inside enterocytes, which did not significantly differ compared to the wild-type strain (*P* > 0.05). The persistence rate was 7.9% for wild-type strain 1711-4, 32.1% for the 1711-4 Δ*escN* mutant, and 8.7% for the complemented 1711 Δ*escN* mutant (Table 3).

### 3.3. T3SS Is Necessary for Enterocyte A/E Lesion Formation and Invasion in the Rabbit Ligated Ileal Loop Model* In Vivo*

**Figure 2:**
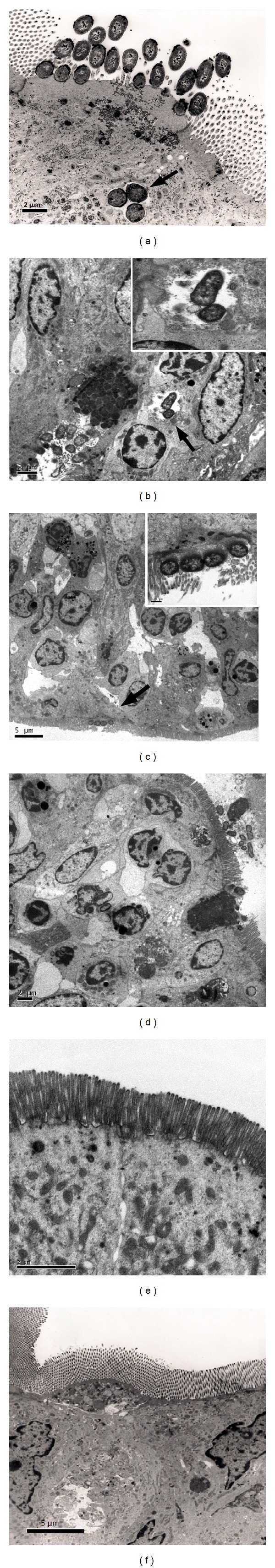
TEM images of rabbit ileal loops infected with aEPEC 1711-4, an isogenic T3SS-mutant or non-pathogenic* E. coli* HS. (a) wild-type strain at 8 h after infection; (b), (c), and (d)—1711-4 wild-type strain at 24 h after infection. Note an epithelial disorganization at 24 h after infection (c) and (d) in tissue infected with aEPEC 1711-4 strain but not 1711-4 Δ*escN* (e) or nonpathogenic* E. coli* strain HS (f). Of note, aEPEC 1711-4 was detected inside an enterocyte at 8 h (a) and 24 h (b) after infection (black arrowheads). Note actin accumulation leading to pedestal formation (black arrow) (c).

To evaluate the interaction of aEPEC 1711-4 with intestinal mucosa* in vivo*, we used the rabbit ligated ileal loop model. Eight or 24 h after infection, wild-type strain 1711-4 was observed intimately attached to the intestinal mucosa with effacement of the microvilli and pedestal formation, which are features of A/E lesions (Figure 2). Eight or 24 h after infection, the wild-type strain was also detected inside enterocytes (Figures 2(a) and 2(b)). An epithelial disorganization was observed 24 h after infection with the wild-type strain (Figures 2(c) and 2(d)). No A/E lesions or invasion was observed with the 1711-4 Δ*escN* mutant (Figure 2(e)) or the nonpathogenic* E. coli* strain HS (Figure 2(f)).

### 3.4. aEPEC 1711-4 Stimulates an Acute PMN Infiltrate in the Rabbit Ileal Loop Model

**Figure 3:**
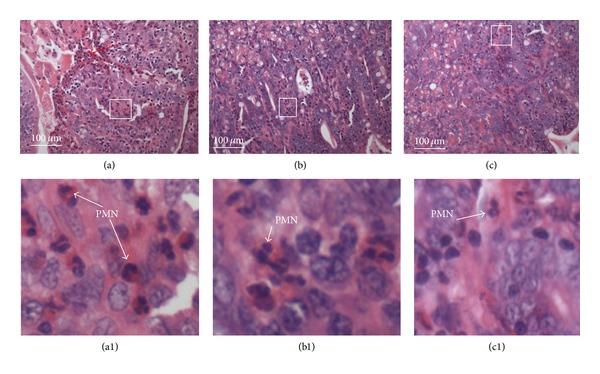
Histopathological analyses of the H&E-stained infected rabbit ileum sections. Squares indicate areas magnified in (Figure 3 (a1), (b1), and (c1)). (a) and (a1)—Ileal loop infected with aEPEC 1711-4; (b) and (b1) ileal loop infected with 1711-4 Δ*escN*; (c) and (c1) ileal loop infected with* E. coli* HS. Note intense intraluminal polymorphonuclear leukocytes (PMN) infiltrate, red arrow, in (Figure 3(a)). Note moderate PMN tissue infiltration in (a1) and (b1).

Sections of the ileum infected with the wild-type aEPEC strain 1711-4 showed a moderate intraepithelial polymorphonuclear leukocytes (PMN) infiltrate, a large number of intraluminal PMN, and intraluminal bleeding (Figure 3(a)-(a1)). The ileum infected with the 1711-4 Δ*escN* had an intraepithelial and intraluminal PMN infiltrate but to a lesser extent than that observed with the wild-type strain (Figure 3(b)-(b1)). In contrast, the ileum infected with the nonpathogenic strain HS showed a discrete polymorphonuclear infiltration (Figure 3(c)-(c1)).

### 3.5. T3SS Is Necessary for Efficient* In Vivo* Translocation of aEPEC 1711-4 in the Rat Model

**Figure 4:**
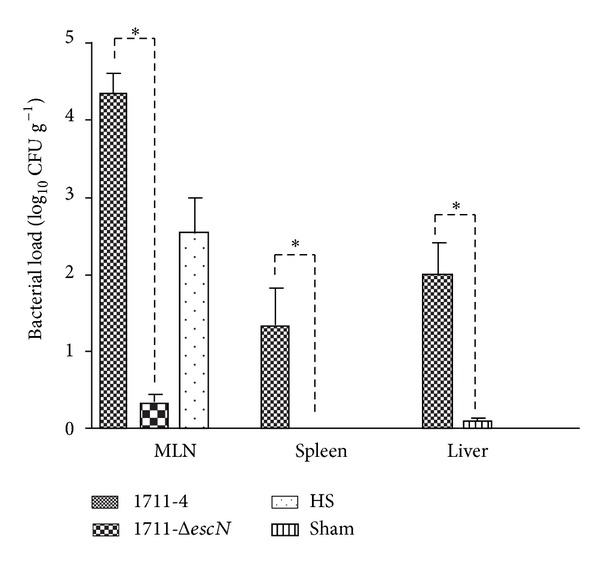
Bacterial translocation assays. Bacterial recovery after BT in mesenteric lymph node (MLN), spleen, and liver 2 h after infection. Statistically significant differences (∗) were observed between aEPEC 1711-4 and 1711-4 Δ*escN* in all compartments (*P* < 0.05). Bacteria were not detected in sham animals. HS strain was only detected in MLN.

The Most striking difference—3 log_10_—was observed in the number of CFU recovered from MLN of animals infected with wild-type strain 1711-4 when compared to that recovered from animals infected with the T3SS-deficient mutant (1711-4 Δ*escN*), but marked reduction in the CFU number was also observed in spleen and liver, since this mutant was not recovered even from these organs (*P* < 0.05) (Figure 4).

## 4. Discussion 

In this study, we analyzed the ability of aEPEC 1711-4 as well as its isogenic mutant deficient in T3SS to adhere to, invade, and persist inside intestinal Caco-2 cells* in vitro*. We also evaluated the ability of these strains to invade and elicit an inflammatory infiltrate in a rabbit ligated ileal loop model* in vivo* and to translocate through the intestinal mucosa in a rat model.

During* in vitro* or* in vivo* interactions, aEPEC strains translocate effector proteins into enterocytes through a T3SS, resulting in the formation of A/E lesions [26]. According to Gauthier et al., 2003, the EscN protein functions as an ATPase, whose absence prevents T3SS assembly, blocking translocation of some structural and effector proteins into the eukaryotic target cell [6]. Our results demonstrated that the 1711-4* escN* mutant was unable to cause A/E lesion in HeLa cells and to adhere effectively to Caco-2 cells thus indicating that T3SS contributes to aEPEC 1711-4 adhesion. This was expected since Tir uses T3SS to reach the eukaryotic cell cytoplasm before inserting into the host cell membrane to serve as an intimin receptor [8]. However, some intimin subtypes have alternative receptors in the eukaryotic membrane [27–29] and flagella also play a role in bacterial adhesion [15, 16, 30]. These previous findings may explain why* escN* deletion decreased but did not abolish the ability of aEPEC 1711-4 to adhere to Caco-2 cells* in vitro.* Additionally, previous studies conducted in our laboratory have demonstrated that aEPEC strains can produce several fimbrial adhesion structures, which could contribute to the adherence process at least in epithelial cells* in vitro* [31].

Considering that aEPEC 1711-4 is able to invade Caco-2 cells and persist and induce IL-8 production, IL-8 secretion by rabbit enterocytes could have driven the polymorphonuclear infiltration observed in ileal loops infected with aEPEC 1711-4 but not with the nonpathogenic* E. coli *HS. On the other hand, some authors reported that an* escN* deficient mutant derived from the tEPEC prototype strain E2348/69 was able to induce very high levels of IL-8 in a flagella-dependent pathway [32, 33]. Consequently, we would expect a more exuberant PMN infiltrate in ileal loops infected with the 1711-4 Δ*escN* strain, but surprisingly the more pronounced intraepithelial and intraluminal PMN infiltrates were observed with the wild-type strain. These data taken together indicate that IL-8 production by enterocytes may not be the main factor determining epithelial infiltration by PMN during aEPEC infection* in vivo*. Another CXC-type chemokine, such as CXCL1 or CXCL5 [34, 35] known to be produced by enterocytes and to have their transcription driven by NF*κ*B signaling pathways triggered via toll-like receptors, may be more important in neutrophil recruitment in the rabbit ileal loop model.

Although the total CFU of the* escN* mutant detected in the intracellular compartment at 48 h was reduced when compared to the wild-type 1711-4 strain, the persistence index (the percentage of intracellular bacteria detected at 48 h) indicated that the 1711-4 Δ*escN *mutant had an increased capacity to persist inside Caco-2 cells (32.1%). Possibly a nonfunctional T3SS reduces the induction of nitric oxide synthase (iNOS) allowing more efficient bacterial persistence, suggesting that iNOS induction may be the result of one or more T3SS-dependent effectors [36]. Persistence may be essential to establish the carrier state, allowing colonization of other parts of the intestinal epithelium by bacteria that escape from infected enterocytes or allowing bacteria to go undetected by phagocytes and antibodies. aEPEC location inside vacuoles with pedestal formation, as demonstrated in HeLa and Caco-2 cells may be another protective factor allowing intracellular persistence [37]. Several studies have shown that* E. coli* is the most commonly isolated pathogen in bacterial translocation events [38–41]. Moreover, some authors have demonstrated the occurrence of bacterial translocation in patients who developed sepsis after surgery [39]. It has been shown that a specific* E. coli* strain isolated from a fatal case of human hemorrhagic pancreatitis was more efficiently translocated to MLNs, blood, and peritoneal fluid [42].

To date, no cases of bacteremia due to EPEC have been described in humans, but strains harboring the* eae* gene have been detected in* E. coli* cultivated from bacteremic neonatal calves [43]. In previous studies by our group, we have shown that some aEPEC strains have the potential to invade and persist in enterocytes (Caco-2 and T84 cells)* in vitro* [16] and to translocate in the rat model [17]. In this study, we demonstrated by electron microscopy that the 1711-4 strain was able to colonize and to form A/E lesions in rabbit intestinal cells in the ileal loop model* in vivo*. This event was not observed with the* escN*-deficient mutant. These results suggest the involvement of T3SS in the translocation event* in vivo*. Liberatore et al. demonstrated that EPEC are able to translocate through the small bowel epithelium and reach not only the MLN but the spleen and the liver as well in a rat model. In this model, translocation of wild-type aEPEC 1711-4 has also been associated with damage to mesenteric microcirculation and hypoperfusion in the liver, small intestine, and kidneys [17]. We demonstrated in this study that a functional T3SS is necessary for efficient bacterial translocation, since a decreased number of bacteria were recovered from the liver and spleen of rats infected with the 1711-4* escN* mutant. Martinez-Argudo et al. reported that T3SS is required for inducing loss of intestinal barrier function and allowing translocation of* Salmonella enterica *strains through M cells [44]. The effector proteins injected via the T3SS that could contribute to driving the translocation events need to be characterized. Although findings in animal models should be extrapolated to humans with caution, our results indicate that aEPEC, an infectious agent theoretically restricted to the intestinal mucosa, has the potential to cross the intestinal barrier under overgrowth conditions which can occur in many clinical situations, such as immunosuppression, antibiotic therapy, biliary obstruction, and other processes that cause changes in the intestinal microbiota.

## 5. Conclusion 

Research carried out with* in vitro* and* in vivo* models have added to our understanding of how bacteria interact with and modify host cells functions leading to the establishment of disease. Our findings indicate that the full virulence potential of aEPEC 1711-4 depends on a functional T3SS, which contributes to efficient adhesion/invasion* in vitro* and* in vivo* and to bacterial translocation to extraintestinal sites.

## Figures and Tables

**Table 1:** Bacterial strains and plasmids used in this study.

Genotype and characteristics	Genotype and characteristics	Source/reference
1711-4	aEPEC O51:H40; wild-type	Gomes et al., 2004 [21]
1711-4 Δ*escN *	*escN*::zeo (Zeo^r^)	This study
1711-4 Δ*escN * (pEscN)	1711-4 Δ*escN *carrying pEscN (Zeo^r^, Clo^r^)	This study
pEscN	pACYC184 carrying the *escN* gene from tEPEC E2348/69 strain	Gauthier et al., 2003 [6]
pKOBEG-Apra^r^	Derivative (Apra^r^) of pKOBEG plasmid encoding the *λ* phage red operon	Chaveroche et al., 2000 [22]
MC4160-*malT*Δ224::*zeo*-(F+)	Source of zeocin cassette	Gift from J. M. Ghigo

^r^Means that the strain is resistant to that antibiotic.

**Table 2:** Primers used for construction and verification of mutation.

Designation	Primer sequence
Allelic exchange	
ESCN.zeo-5	5′-TGGGAATAATATCGAACTTAAAGTATTAGGAACGGTAAATGGTCATCGCTTGCATTAGAAAG-3′
ESCN.zeo-3	5′-CGCTCTGCTTTTACGAATAGATAAAATTCTGTCCAACATATTCAGAATGATGCAGAGATGTAAG-3′
Verification	
ESCN.verf-5	5′-TCAGGCGCTATGTGAAGAAA-3′
ESCN.verf-3	5′-TACGCCTGCTTAGAGGCAAT-3′

^a^Underlined bases correspond to 5′ and 3′ regions of the *Sh ble* gene, which encodes zeocin resistance.

**Table 3:** Intracellular bacteria at three and 48 hours after infection of Caco-2 cells.

Strain	Number of intracellular bacteria at 3 hours (mean ± SD)	Number of intracellular bacteria at 48 hours (mean ± SD)	Mean bacterial persistence index
1711-4 WT	41,666 ± 16,093.5	3,333 ± 1,310.9	7.9%
1711-4 Δ*escN *	1350 ± 129.1	433 ± 110.1	32.1%
1711-4 Δ*escN* (pEscN)	37,500 ± 3,535.5	3,266 ± 503.3	8.7%
